# Unobstructive Body Area Networks (BAN) for Efficient Movement Monitoring

**DOI:** 10.3390/s120912473

**Published:** 2012-09-13

**Authors:** Filipe Felisberto, Nuno Costa, Florentino Fdez-Riverola, António Pereira

**Affiliations:** 1 Higher Technical School of Computer Engineering, University of Vigo, Polytechnic Building, Campus Universitario As Lagoas s/n, 32004 Ourense, Spain; E-Mail: filipe.felisberto@ipleiria.pt; 2 INOV INESC INNOVATION, Institute of New Technologies of Leiria, P-2411-901, Leiria, Portugal; E-Mails: nuno.costa@ipleiria.pt (N.C.); apereira@ipleiria.pt (A.P.); 3 Computer Science and Communications Research Centre, School of Technology and Management, Polytechnic Institute of Leiria, P-2411-901, Leiria, Portugal

**Keywords:** Wireless Body Area Networks, motion recognition, rehabilitation, profiling, inertial and physiological sensors

## Abstract

The technological advances in medical sensors, low-power microelectronics and miniaturization, wireless communications and networks have enabled the appearance of a new generation of wireless sensor networks: the so-called wireless body area networks (WBAN). These networks can be used for continuous monitoring of vital parameters, movement, and the surrounding environment. The data gathered by these networks contributes to improve users' quality of life and allows the creation of a knowledge database by using learning techniques, useful to infer abnormal behaviour. In this paper we present a wireless body area network architecture to recognize human movement, identify human postures and detect harmful activities in order to prevent risk situations. The WBAN was created using tiny, cheap and low-power nodes with inertial and physiological sensors, strategically placed on the human body. Doing so, in an as ubiquitous as possible way, ensures that its impact on the users' daily actions is minimum. The information collected by these sensors is transmitted to a central server capable of analysing and processing their data. The proposed system creates movement profiles based on the data sent by the WBAN's nodes, and is able to detect in real time any abnormal movement and allows for a monitored rehabilitation of the user.

## 1. Introduction

The technological advances in the MEMS (Micro-Electro-Mechanical Systems) and CMOS (Complementary Metal-Oxide-Semiconductor) areas have led to the appearance of low cost and low power wireless communications, which in turn enabled wireless sensor networks (WSN). These kind of networks opened new applications areas in fields such as military surveillance, environmental monitoring, and intrusion detection, among others.

One area which has gained lots of attention from academia and commercial sectors is health monitoring. With the appearance of biomedical sensors and suitable network protocols, a new generation of wireless sensor networks have emerged, the so-called wireless body area networks (WBANs). These networks can be used for continuous monitoring of vital parameters, movement, and the surrounding environment. The data gathered by these networks contributes to improve users' quality of life and allows creating useful knowledge bases. The work described in this article, had as main objective to exploit the previously developed technologies [1] to create a viable solution for human motion analyses.

The motivation behind such objective came from the problems encountered while developing a fall detection system; while the developed system was able to precisely detect normal falls (by normal falls we mean falls that are not hampered in any way, and the person hits the floor without intermediate deceleration) while using fewer resources than preceding projects, the detection still failed when there was an intermediate deceleration, caused, for example, by the person trying to avoid the fall by clinging to something. This system was also unable to distinguishing extreme but harmless activities (e.g., sports) from harmful ones (e.g., falling down stairs).

Our proposal for solving the presented problem is through the use of motion profiles, created using the data acquired from the WSN. Using these profiles it is expected that it will be possible not only to more efficiently detect normal falls, but also, those falls that were previously undetected, like hampered falls or those caused by sudden onset diseases (for example a stroke). In addition, by using intelligent agents constantly analyzing the variations of the profiles, it will be possible to help diagnose physical and posture deterioration that tend to cause accidents [2].

During a preliminary study [3] it was concluded that the analysis of human movement is one of the monitoring tasks that require more resources. As such, it often becomes impractical to do motion analysis and other types of physiological data analysis at the same time, thereby isolating the motion analysis. This article proposes a solution for this problem, creating the foundation for a complete system for human monitoring.

By focusing on the detection of abnormal movements and the monitoring of the users' postures instead of complete motion tracking, the proposed system can have low energy consumption and be almost ubiquitous, enabling it to be used daily. This allows the system to go beyond detecting only present problems to being able to, for example, do long term patient rehabilitation or detect any deterioration in the users's gait.

The remainder of this paper is organized as follows: Section 2 overviews the related work on wearable body area networks with special focus on applications related with in hospital and disaster events, residential healthcare monitoring, and motion and activities. Section 3 describes the system architecture and platform used to do movement recognition. Next, Section 4 presents the executed tests and their results in order to assert the viability of the proposed movement recognition platform and finally Section 5 draws the conclusions and highlights ongoing work.

## 2. Related Work

This section gives an overview of related Body Area Networks projects. Although BANs support several applications areas, such as sports, fitness, entertainment, *etc.*, here we focus on health care applications, they being experimental platforms, ready to use solutions or even finished products.

Focusing on the sensor nodes we, generically, can characterize a Body Area Network as implantable or wearable. In implantable BANs sensor nodes are placed under the skin of the patient, while in wearable BANs the sensor nodes are embedded in clothes or near the skin surface. Wearable BANs can also be split into wired and wireless, according to the type of medium used to connect sensor nodes. For specific information on implantable and wearable technologies used in WBANs application see [4,5].

Considering the genesis of the prototype described in this article, this section focuses on wireless wearable BANs projects in three domains: (i) in hospital and disaster events, (ii) residential healthcare monitoring and (iii) motion and daily living activities, although the solution presented in this article is more suitable for residential healthcare monitoring and motion monitoring.

### 2.1. In Hospital and Disaster Events

The first set of existing projects presented here belongs to the hospital and disaster events domains. These projects aim to facilitate and even accelerate the response to be provided to patients by enhancing the triage process, for example, and on the other hand, gather data at disaster sites and notify target hospitals about the patients' conditions even before patients arrival at the hospital.

Code Blue project [6] is probably one of the most referenced projects in the wireless BAN area. It employs *ad hoc* networks of off-the-shelf motes and medical sensors such as ECG, SPO_2_, motion and EMG, in order to give a response in pre-hospital and in-hospital emergency care, disaster response and consequent triage process and stroke patient rehabilitation. Code Blue has adopted a publish/subscribe data delivery model where rescue and medical personnel subscribe to data of interest. Code Blue also includes a system called MoteTrack [7], useful for calculating the 3D location of patients, nurses, physicians or medical equipment.

The same authors of Code Blue are also involved in a related project called Mercury [8] which aims to support high-resolution motion studies of patients with Parkinson's disease, stroke, and epilepsy through the use of accelerometers, gyroscopes, and/or physiological sensors, hence targeted to clinical applications.

LiveNet [9] allows people to receive real-time information about their health status and can also communicate with care givers. It also embeds a learning infrastructure based in statistical machine learning techniques in order to distinguish shivering from normal body movements. The authors found that some types of shivering are very close to the body temperature and also argue that LiveNet could classify the movement states of Parkinson's patients and identify an epileptic seizure in epilepsy patients. LiveNet adopted a wired approach for sensor node connection.

### 2.2. Residential Healthcare Monitoring

Another application area of the healthcare BANs is residential healthcare monitoring. This application domain is very important and strategic as in the most case it frees beds in hospitals once patients can be returned to their homes while still being monitored by the medical staff.

ALARM-NET [10,11] is a home healthcare system that integrates environmental and physiological sensors in a scalable and heterogeneous architecture. ALARM-NET includes an analysis program called Circadian Activity Rhythm (CAR) [11] which processes sensor data for learning individual behavior patterns.

The BASUMA project [12] seeks continuous health monitoring of chronically ill patients in their own homes in order to detect when the health state changes to worse. It can alert and recommend actions in a timely manner before critical conditions occur. For further information on specific sensing technology for individuals with chronic diseases see [13].

### 2.3. Motion and Activities

Another application area of wireless BANs in the healthcare field is the monitoring of movements and everyday activities. To achieve this wearable sensors with high resolution are usually used. For a detailed review of sensing components utilized for body motion measurement see [14].

The University of Washington and Intel Research Seattle developed a prototype to identify people's activities [15]. The system relies on Radio Frequency Identification (RFID) tags attached to everyday objects which are tracked using tag readers embedded in gloves and the activities are inferred using sequences of tag readings.

The work that is being done at the Center for Future Health of the Medical Center of University of Rochester [16] is very close to ours. Like us, the researchers want to answer to the question “What can be learned about an individual's health state by observing the motion, activity and interactions in one's natural environment?” [16]. The objective is to learn what is “normal” for the person and to detect and monitor trends that may indicate developmental or incipient health issues [16], and hence detect such conditions in the earliest possible stage. Like our solution, this project employs inertial sensors for movement research and collects vital signs in order to complete the clinic information of the person and no further information is available.

In [17] its authors proposed a wireless BAN composed of off-the-shelf sensor platforms and a PDA, as a personal server, capable of monitoring user physiological state and computer-assisted physical rehabilitation (such as for ankle injuries).

In [18] a telerehabilitation platform which includes two H264 videoconferencing CODECs (Tandberg 550 MXP) with integrated wide-angle view cameras, remote-controlled pan, tilt zoom (PTZ), 20-inch liquid crystal display (LCD) screens and a dedicated modular software interface for user-friendly control of videoconferencing connections and PTZ cameras is described. The authors employ user-friendly interfaces in order to insure that interactions between clinicians and clients during the telerehabilitation sessions were accepted. Finally in [19] a wireless BAN in conjunction with a machine learning algorithm to classify movements and register the starting and finishing instant of each movement is presented. To the best of our knowledge, this is the closest project to ours as both share the same aims through the use of the same type of sensors. This project relies on triaxial accelerometers attached to Imote2 [20] sensor nodes.

## 3. General Unobstructive BAN Architecture

The similarities and differences between our proposal and the currently existing ones can be summarized in two major aspects: (i) the use of individual profiles and (ii) the hardware used on the sensor node platform.

Our proposal is based on profiles automatically created using learning techniques. While the profiles all start from a common model created from a normalized user profile (like in [16]), they evolve based on each user's different activities and movements, making them unique for each user.

These profiles are then used by the system to infer problems, instead of relying solely on interpretation of real-time sensed data (deterministic approach) [8,12,17]. They are also used to infer deviations from the normal user condition unlike ALARM-NET CAR [11] which uses individual behavior patterns for aiding of context-aware power management and privacy.

By using learning techniques and auto-built user profiles our project intends to be more autonomous, in order to avoid technical intervention configuring parameters for each user that uses the system, like those necessary in [18].

In terms of hardware, we developed our own sensor node so that our platform could be small, lightweight and very responsive. In [19] the Imote2 sensor node which is power hungry and has dimensions that can compromise comfort was used, also their solution uses only accelerometer data while our solution uses data from accelerometer, gyroscopes and magnetometers which enables a more precise reconstruction of human movement. For sensor node connection we opted for using wireless communication instead of a wired approach [9] as it compromises the flexibility and scalability of the system.

**Figure 1.**
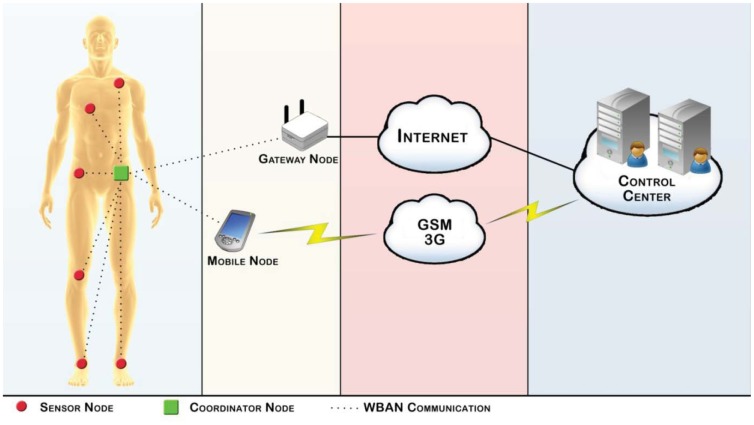
WBAN motion architecture.

Finally, our proposal is not targeted to explicitly identify user routines [15], but identify movements and postures in order to raise alarms. Therefore, the work presented in this paper intends to use a technological solution to recognize human movement, identify human postures and detect harmful activities in order to prevent risk situations. To achieve this, tiny sensors nodes with wireless communication, computational and energy harvesting capabilities are networked around the human body forming a wireless body area network called, in our proposal, WBAN Motion. Figure 1 illustrates our architecture proposal that comprises five basic components:

- Sensor Node: Responsible for acquiring data of inertial and physiological sensors and transmitting them to the Coordinator Node. The sensor node has, if necessary, actuation capabilities and both its hardware details and body placement are described in the next subsections;
- Coordinator Node: Responsible for serving as a forwarder of data gathered by the Sensor Nodes; it forwards the data to the Gateway Node or to the Mobile Node and can do some kind of preprocessing before referral. This type of node can have inertial and physiological sensors, acquire and transmit their data to the Gateway Node or Mobile Node;
- Gateway Node: It is the interface between the WBAN and the network, that provides the Internet connection;
- Mobile Node: An alternative interface used when the Gateway Node or Internet connection are not available;
- Control Center: Responsible for the registration and post processing of the motion events sent by de Sensor Nodes.

The Sensors Nodes are connected to the Coordinator Node, in a wireless star topology, creating a short range wireless body area network. Inertial and physiological sensors are spread over strategic human body locations in order to gather raw data which is then directly forwarded to the Coordinator Node. The Coordinator Node is a responsible for data fusion and the transmission to the Control Center. This transmission can be made through to Internet or by GSM or a 3 G network according to their availability. The Internet is the preferred connection, but when the Gateway Node is unavailable, the Coordinator Node can use a Bluetooth module to communicate directly with a Mobile Node running a bridge application.

When data arrives at the Control Center it is processed and used to automatically create the user profile by using learning techniques. Once the profile is created the system can already use intelligent approaches to infer abnormal behaviors. When an abnormal situation is detected the system will take actions according to the specific application (rehabilitation, posture, correct movement, *etc.*).

The WBAN Motion architecture was designed to meet the requirements of unobstructiveness and scalability, which means that more Sensor Nodes can be added on the fly for more system accuracy or different acquired signals without compromising the user comfort.

### 3.1. Sensor Node

**Figure 2.**
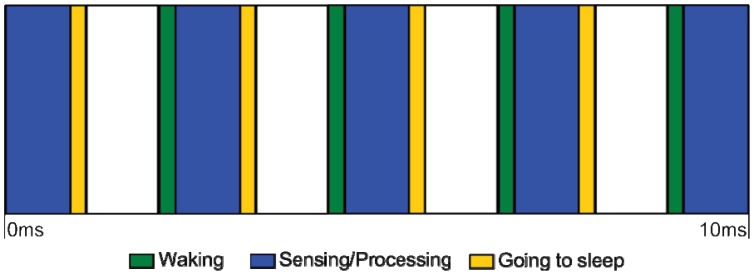
Windows of active and sleep states.

One important conclusion arising from our previous work on fall detection, was the necessity of maximizing the time the system spends in sleep mode. Even if the time spent collecting and processing each batch of sensor data may be small, if we add the time spent going to sleep and coming back from it to the fact that we need an high sampling rate to correctly record human movement [21], the system ends up spending more time in active mode than in sleep mode, as it can be observed in Figure 2.

For the fall detection purpose this was solved by using statistical methods to reduce the sampling to less than half the normally used rate and all the more demanding tasks were transferred to the command center.

**Figure 3.**
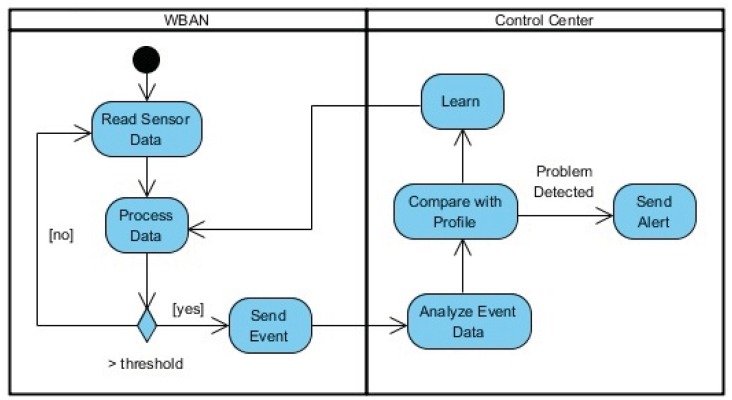
Sensor node data flow.

Here, the same solution cannot be directly applied. While an unhampered fall is an isolated event that can be detected only by event information, the same cannot be used to detect hampered falls, diagnose posture deterioration and detecting incorrectly done activities as it requires a continuous analysis of the body's movement. Figure 3 depicts a diagram of the necessary information flow.

For movement recognition there is the added problem that using only accelerometers is not a viable solution, as this would lead to a high bias. This problem is not corrected by only adding a gyroscope [22], it is necessary to also use a magnetometer [23]. This brings a second problem to the equation, the added processing necessary to fuse the data from these three different kinds of sensors. Any of these problems by itself can put a big strain on the system's autonomy. Both problems at the same time and even a normal task, like sending and receiving data, and system may end up discarding information due to not being able to keep up.

In order for the node to be able to apply advanced filters without compromising the remaining activities it was necessary to use a more advanced 32 bit microcontroller, rather than the more commonly used 8 bit one [24], but this option also meant that during the active phase the power consumption would be higher, leading to lower autonomy.

**Figure 4.**
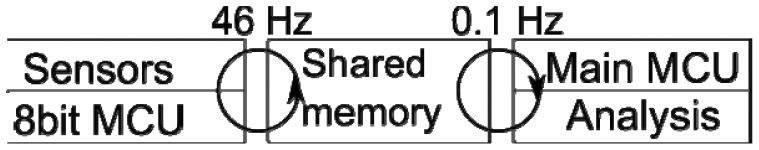
Microcontroller Unit's (MCU) diagram.

Our solution for this problem was to develop a Sensor Node which uses an ultra-low power and basic microcontroller for the collecting tasks while the main microcontroller is responsible for the normal system activities and for processing the sensor data, doing this last task in batches of data instead of for every sample. Figure 4 contains a diagram explaining the process of data gathering and analysis done by the node. The 8 bit MCU gathers data from the sensors 46 times per second, storing it in the shared memory. Once each 10 seconds, the data stored in the shared memory is all read by the Main MCU for subsequent analysis.

In terms of sensors, we opted to use a system-in-package solution like the LSM333D from ST [25]. This drastically reduces both the node size and the energy consumption, as all the three sensors are in the same “chip”. Also, by using this type of solution we reduce the problems in sensor calibration minimizing the problems described in [26].

### 3.2. Node Placement

One thing that one learns soon after starting to work on pervasive healthcare systems is that if the system is cumbersome to use, unesthetic or hard to maintain it will not be accepted, even if it is one hundred percent accurate. So, while it would be preferable to have a wide array of nodes spread throughout the user's body, in order to precisely recreate the entire movement using known motion capture techniques [27,28], it was imperative to define what parts of the human body need to be directly monitored and those whose movement can be extrapolated (*i.e.*, if two parts are connected by a joint and one is being directly monitored the other part's position and movement can be estimated).

Depending if the objective of the WBAN is to detect and prevent or to help during rehabilitation there is a different number of nodes that need to be used. For the rehabilitation domain it is only necessary to measure the parts that are involved in the rehabilitation itself. These extra nodes need only to be added when necessary and take advantage of the scalability of our architecture. This task can be easily accomplished by the user himself not requiring any type of technical knowledge to do so.

On the other hand, to monitor the human gait it is necessary to measure multiple variables. This requires a big number of sensors which, at this time, is not viable in a day-to-day basis. What was decided was to use an initial study phase, where all the different parts of the body are recorded, with the objective of creating a preliminary motion profile and to discover if there is an existing problem with the user's gait. After the motion profiles are created, it is then possible to drastically reduce the number of points from where the data is collected. This is only possible because what we want to monitor is variations in the gait and detect sudden and irregular movements and not record what the user does.

**Figure 5.**
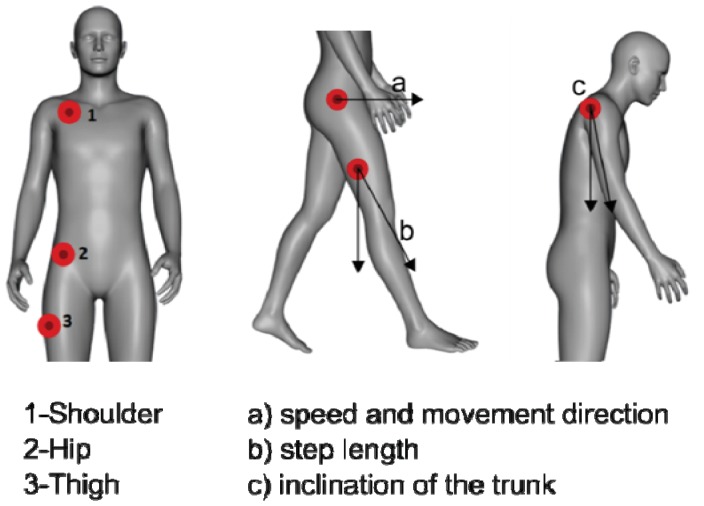
Nodes' placement.

During the monitoring phase, the points that are being directly recorded are the shoulder, hip and thigh areas. The shoulder area and hip were selected due to their relevance on the gait cycle. The inclination of the shoulders can drastically influence the center of mass during walking and different ADLs, leading to a higher chance of falling. The thigh area can be used to analyze the walking patterns specially to identify variations on the step length and speed. The node placed in the hip area is the coordinator node of the entire architecture so it is not an additional node that is being used but at the same time its position is also one that is important to measure as it is very close to the body's center of mass which means it is a very good place to measure acceleration both to detect impact and to define a movement reference for the other nodes. The nodes' placement is visually explained in Figure 5.

## 4. Tests and Results

To be able to assert the viability of our movement recognition platform, it was necessary to test its premises using a realistic testing scenario. To this end, was created a new testing platform and defined a series of tests.

### 4.1. Experimental Platform

**Figure 6.**
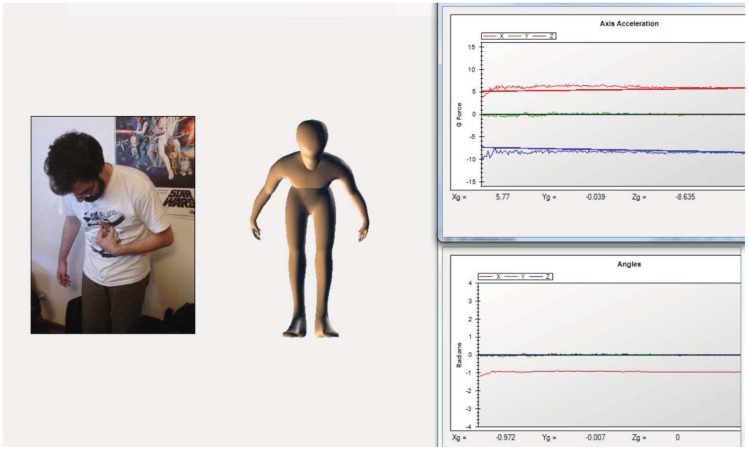
Testing software package.

To test if it would be possible to have an illustrative representation of the human movement by monitoring only a restricted number of the body points, we have created a testing software that applied the transformation matrix, obtained by fusing the WBAN sensorial data, to a Computer Generated Human Body (CGHB). This software, depicted in Figure 6, was developed with the Microsoft XNA API. The XNA SDK real time manipulation of the 3D models is done through the skeletal animation technique, which allows for a more direct application of the data returned by the WBAN.

For the WBAN side, a new sensor board containing an ultra-low power 8 bit PIC(tm) microcontroller, a gyroscope, a magnetometer and an accelerometer was developed. This new sensor board differs from the more commonly used sensor boards for the more active form in which it works. Normally it is the main board that collects the data from the sensor boards but in this case the sensor board is the one responsible for collecting the data and storing it in the main board's shared memory.

### 4.2. Data Collection and Experimental Setup

The platform's testing was divided into three separate testing phases, each with an increased complexity. The first testing phase consists of data being collected from a single point on the human body. This data is then directly applied to the CGHB using only simple sensor fusing formulas and without any kind of filter being applied to the received data. What is being measured, in this phase, is how realistically the CGHB can mimic the movement from the specific part of the body whose data is being collected and how it affects the representation of the non-monitored parts.

In the second testing phase, we started using sensor nodes on the three aforementioned motorization points (thigh, hip and shoulder). During this phase the data is still directly applied to the CGHB as what is being studied is how precise can the system work without any kind of data processing.

The third phase uses the same number of sensor nodes, but this time, before the data is applied to the CGHD, it is filtered in order to remove as much noise as possible. For these tests were implemented simple versions of the Kalman filter (KF), as well as, more advanced versions of the extended Kalman filter (EKF) specially designed for fusing data from inertial sensors [29,30].

To guarantee the uniformity of the testing phases, the data used was initially recorded instead of working with live data, and the dataset used on all three testing phases was the same. In order to verify the collected data, all the testing sessions were recorded so the CGHB animations could be compared to the actual human movement.

The types of the tests recorded spanned from simple movements of isolated parts of the body, to more complex actions like sitting down and picking up objects from the ground. In terms of actual sensorial data and its conversion to speed, heading and attitude, three different approaches were tested.

In the first, which served as control, speed was calculated by simply integrating the acceleration returned by the accelerometer and the orientation by integrating the angular speed returned by the gyroscope, both without using any error compensation technique. There was no fusion of sensorial data, so the heading was not obtained nor was the magnetometer data used.

The second approach consisted on fusing the normalized data from the three sensors using a Direction Cosine Matrix (DCM) algorithm to calculate the orientation and heading. Moreover, the normalized accelerometer data was also filtered using a KF before being integrated to calculate the movement speed. Finally, the third final approach used an EKF for sensorial data fusion.

### 4.3. Results and Discussion

While describing the results we will differentiate the movement of single points of the body and while the body itself stays stationary (e.g., raising an arm, bending the trunk and moving a leg) from a full body movement (e.g., walking and running).

The first testing phase presented satisfactory results for the representation of rotation and single point movement. As expected, there was vibration on the CGHB due to the noise in the data but movements were correctly mimicked. Full body movement was not tested during this initial phase.

**Figure 7.**
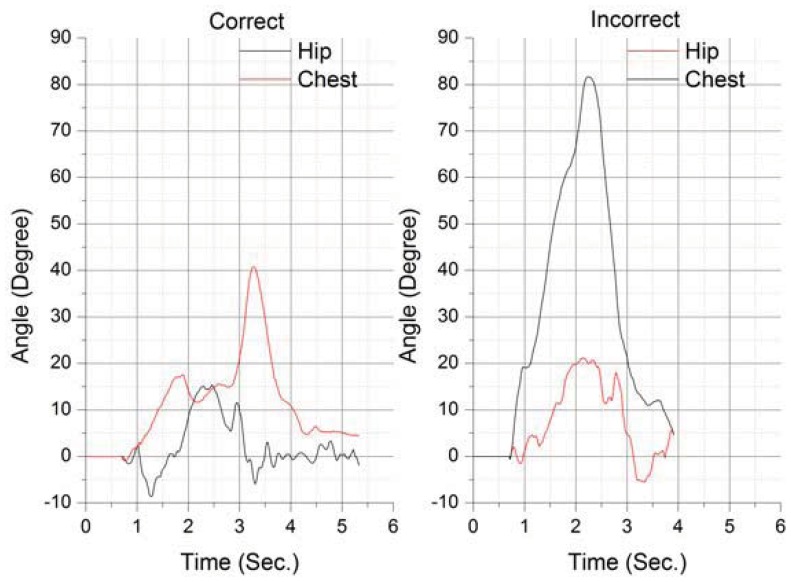
Comparing the data collected during a correct and an incorrect pickup action.

Using more data sources it was possible to recognize different full body actions like, for example, to differentiate the correct picking up of an object from the incorrect way. In this context, Figure 7 shows a graph created using the data from two of the runs of the picking up test.

**Figure 8.**
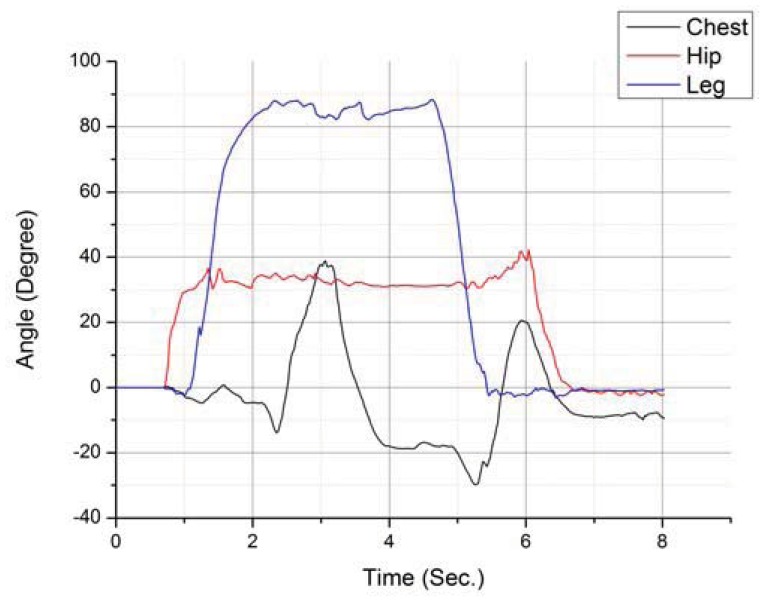
Comparative study of filters applied to the hip's pitch rotation.

Another action whose identification is greatly helped by using multiple sensors is sitting down. Sitting down is one of the hardest ADLs to distinguish from a fall, so being able to correctly identify it is very important. Figure 8 presents a graphical representation containing a test run corresponding to a sit down action.

**Figure 9.**
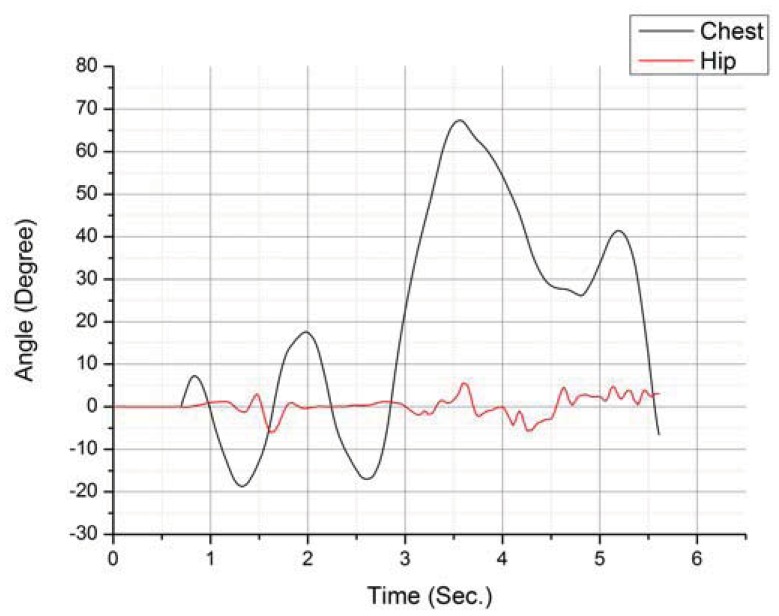
Comparative study of pitch rotation during a normal walk recorded on the chest and on the hip.

During the second phase, while there was more information which enabled more human like body movements, the added noise from the two extra data sources meant that these movements were also more inconsistent and the body's vibration became even more noticeable. For full body movement only the speed and direction vector from the hip sensor node was considered. This was decided to avoid having to spend processing power filtering and compensating for the changes in orientation of the torso. Regarding this issue, Figure 9 depicts the user walking in a straight line while moving his torso.

The third stage showed how important it is to apply filtering when processing movements using only inertial data. The jitter in the animations was reduced to a minimum, enabling for a very precise representation of the actual movement being recorded. Slow and normal full body movement was also successful mimicked by the CGHB. On the other hand, during rapid movements the representation was far from perfect and after these actions had ended, the error still propagated to the following activities.

**Figure 10.**
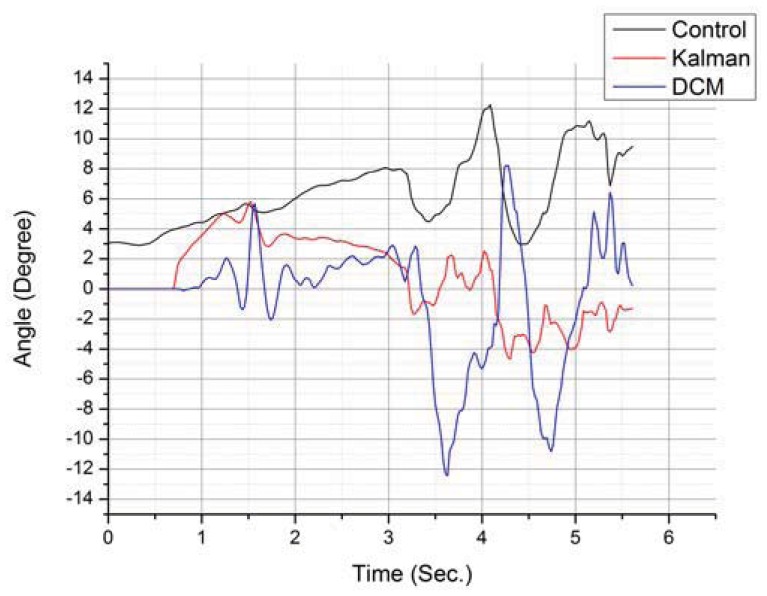
Study of the three different approaches for calculating orientation.

Figure 10 shows a comparative study of the three different approaches to sensor data processing, the recoded data is from a walking test with as few oscillations as possible. The filter that obtains better performance was the EKF, confirming the results of comparing the CGHB animation with the video of the recorded test.

**Figure 11.**
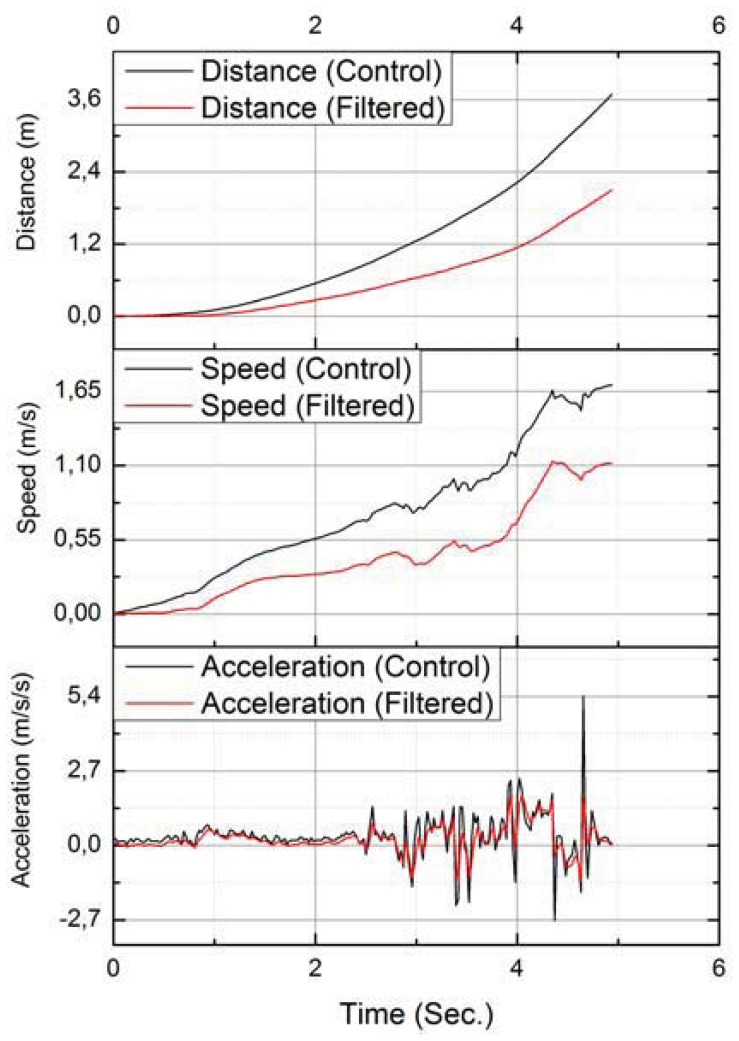
Distance estimation with and without filtering.

An important issue is that if the error is not effectively corrected, it will propagate and increase to a point where it becomes impossible to identify which action was performed. This behavior can be easily observed in even a simple action like walking in a straight line. Figure 11 shows the data recorded from a user walking in a straight line for two meters. The distance calculated without filters was 3.684 meters and with filtering 2.097 meters. This represents a 45.71% error for the unfiltered data and only 4.62% for the filtered data.

## 5. Conclusions

This paper presents a technological solution to recognize human movement, identify human postures and detect harmful activities in order to prevent risk situations. To achieve this, sensors nodes with wireless communication, computational and energy harvesting capabilities are networked around the human body forming a wireless body area network (WBAN).

The WBAN is created using developed tiny, cheap and low-power nodes with inertial and physiological sensors, strategically placed on the human body. The information collected by these sensors is transmitted to a Control Centre capable of analysing and processing their data. The proposed system creates the movement profile based on the data sent by the WBAN nodes, is able to detect any abnormal movement and allows for a monitored rehabilitation of the user.

The system evaluation was based on three scenarios. From the results gathered we were able to conclude that, for our objectives, three nodes placed on different body areas (one in the hip, another in the shoulder and one in the thigh) are enough for correct motion recognition.

As a future work, we intend to develop different types of Beliefs-Desires-Intentions (BDI) agents in order to automate the task of detecting abnormal movement patterns following a federated approach. The multi-agent system will combine deliberative agents together with CBR techniques for improving their learning capabilities [31]. The operative platform will be implemented using the JADE [32] agent framework, taking advantage of its built-in FIPA [33] standard interaction protocols. As a JADE-based multi-agent system, the server side will be easily distributed among several machines, where each agent will run in a different node, even on a different network.
